# Observing How Glutathione and S-Hexyl Glutathione Bind to Glutathione S-Transferase from *Rhipicephalus* (*Boophilus*) *microplus*

**DOI:** 10.3390/ijms232112775

**Published:** 2022-10-23

**Authors:** Warin Rangubpit, Eukote Suwan, Danai Sangthong, Kannika Wongpanit, Roger W. Stich, Prapasiri Pongprayoon, Sathaporn Jittapalapong

**Affiliations:** 1 Kasetsart Vaccines and Biologies Innovation Centre, Kasetsart University, Bangkok 10900, Thailand; 2 Department of Veterinary Technology, Faculty of Veterinary Technology, Kasetsart University, Bangkok 10900, Thailand; 3 Department of Chemistry, Faculty of Science, Kasetsart University, Bangkok 10900, Thailand; 4 Department of Agriculture and Resources, Faculty of Natural Resources and Agro-Industry, Chalermphrakiat Sakon Nakhon Province Campus, Kasetsart University, Sakon Nakhon 47000, Thailand; 5 Department of Veterinary Pathobiology, University of Missouri, Columbia, MO 65211, USA; 6 Center for Advanced Studies in Nanotechnology for Chemical, Food and Agricultural Industries, KU Institute for Advanced Studies, Kasetsart University, Bangkok 10900, Thailand

**Keywords:** glutathione s-transferase, glutathione, s-hexyl glutathione, MD simulation, *R. microplus*

## Abstract

*Rhipicephalus (Boophilus) microplus* is one of the most widespread ticks causing a massive loss to livestock production. The long-term use of acaracides rapidly develops acaracide resistance. In *R. microplus*, enhancing the metabolic activity of glutathione S-transferase (RmGST) is one of the mechanisms underlying acaracide resistance. RmGST catalyzes the conjugation of glutathione (GSH) to insecticides causing an easy-to-excrete conjugate. The active RmGST dimer contains two active sites (hydrophobic co-substrate binding site (H-site) and GSH binding site (G-site)) in each monomer. To preserve the insecticide efficacy, s-hexyl glutathione (GTX), a GST inhibitor, has been used as a synergist. To date, no molecular information on the RmGST-GSH/GTX complex is available. The insight is important for developing a novel RmGST inhibitor. Therefore, in this work, molecular dynamics simulations (MD) were performed to explore the binding of GTX and GSH to RmGST. GSH binds tighter and sits rigidly inside the G-site, while flexible GTX occupies both active sites. In GSH, the backbone mainly interacts with W8, R43, W46, K50, N59, L60, Q72, and S73, while its thiol group directs to Y7. In contrast, the aliphatic hexyl of GTX protrudes into the H-site and allows a flexible peptide core to form various interactions. Such high GTX flexibility and the protrusion of its hexyl moiety to the H-site suggest the dual role of GTX in preventing the conjugation reaction and the binding of acaracide. This insight can provide a better understanding of an important insecticide-resistance mechanism, which may in turn facilitate the development of novel approaches to tick control.

## 1. Introduction

Ticks are blood-sucking ectoparasites that can transmit etiologic agents of human and animal diseases and cause enormous loss to global livestock production [1]. To control these notorious parasites, acaracides are a conventional frontline tool [2]; nonetheless the fast development of acaracide resistance significantly compromises the efficacy of acaracides and threatens current and future tick control. Acaracide resistance in ticks has become a major problem globally. Currently, ticks have exhibited resistance to several pesticides such as organochlorines [3], organophosphates [4], amidines [3,5], synthetic pyrethroids [3,4,5], macrocyclic lactones [4,5], and phenylpyrazoles [4]. Such acaricide failures generate important economic losses in cattle production around the world [6]. Effective strategies to prevent or conquer tick resistance have become urgently needed.

*Rhipicephalus* (*Boophilus*) *microplus*, the southern cattle tick, is one of the most widespread invasive ticks worldwide [7]. *R. (B.*) *microplus* tick is a pathogen vector that causes babesiosis (*Babesia bovis* and *Babesia bigemina*) and anaplasmosis (*Anaplasma marginale*), which are the most impactful tick-borne diseases of cattle, globally [8]. Pesticides serve as the primary strategies for *R.* (*B.*) *microplus* control; however, rapid resistance to such chemicals in *R.* (*B.*) *microplus* has been reported [9]. *R.* (*B.*) *microplus* was reported to be resistant to many common pesticides such as organophosphate, pyrethroid, fipronil, amitraz, and macrocyclic lactone [9]. Enhancing the metabolic activity of detoxification enzymes such as cytochrome P450, esterase, and glutathione-S-transferase (GST) is one of mechanisms underlying chemical resistance in *R. microplus* [9,10,11,12,13]. *R. microplus* glutathione S-transferase (RmGST) receives more attention due to its involvement in tick resistance [14]. RmGST was found to play a critical role in cellular detoxification against xenobiotics such as acaracides [15]. RmGSTs were reported to be involved in organophosphate, organochloride, and pyrethroid resistance [14,16]. To date, the combination of pesticide and synergist was suggested to be effective against metabolic resistance [17,18]. Thus, GST inhibitors as synergist to preserve the insecticide efficacy are used. S-hexyl glutathione (GTX) is the known GST inhibitor which is widely used as a synergist due to its ability to inhibit many insect GSTs and vertebrate GSTs [19,20,21]. In order to use GTX as a synergist in *R. microplus* tick, it is necessary to unravel the binding mechanism of GTX to RmGST. 

**Figure 1:**
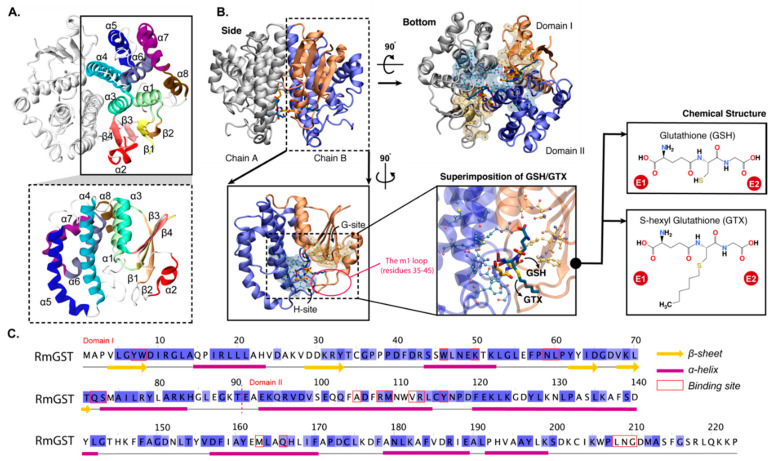
(**A**) Cartoon views of *R. (B.) microplus* GST (RmGST) with labelled secondary structure (α1−α8  and β1−β4). (**B**) Side and bottom views of RmGST homodimer (chains (**A**,**B**)). Each subunit contains domains I (orange) and II (blue) where the mu loop (m1) is shown in a red circle. The ligand-binding sites (G- and H- sites) with bound GSH (yellow) and GTX (dark cyan) are shown as transparent yellow and blue surfaces. The chemical structures of both ligands are shown on the right where their termini are defined as “E1” and “E2”. (**C**) Sequence alignment of RmGST where the secondary structure and residues in the binding site are labelled.

In mammals, GSTs are classified into five families, namely alpha (α), mu (μ), pi (π), theta (θ), and sigma (σ), based on their sequence similarity and cross-immunoreactivity [10]. The *R.* (*B.*) *microplus* GST (RmGST) was reported to be closely related to the μ-class GST [8,22]. GSTs are multifunctional enzymes that protect cells against chemical toxicity and oxidative stress [23], thus contributing to detoxification of acaricides [24]. GSTs detoxify toxic agents or insecticides by catalyzing the conjugation of glutathione (GSH) to xenobiotic which causes a resulting conjugate that is more soluble and easier to excrete from the cell [14,25]. In *R. microplus*, RmGST contains 223 amino acids. The N-terminus displays higher sequence similarity than the C-terminus, which is common throughout GST families [26]. The active form of RmGSTs is a dimer. Each monomer contains eight helixes (α1−α8) and four beta-strands (β1−β4) (Figure 1A). Each subunit (chains A and B) has two domains: domains I (N-terminal) and II (C-terminal) (Figure 1B). The N-terminal domain (I) is rather conserved, while domain II is variable [27]. Two active sites (H- and G- sites) are identified in each monomer. The highly conserved G-site (GSH binding site) is in domain I, while domain II holds the diverse H-site (hydrophobic co-substrate binding site) (Figure 1B). RmGST also contains the “mu” loop (m1) (residues 35–40) like other μ-class GSTs (Figure 1B) [8,22,28]. This loop was reported to be one of the unique features specific to μ-class GSTs [28].

To date, several GST-GSH/GTX crystal structures have been solved [19,29,30,31,32], but no molecular information on RmGST-GSH/GTX complexes is available. Such data are crucial for understanding how RmGST responds to GTX in comparison with its natural substrate GSH. In order to use GTX inhibitor as a synergist and develop novel RmGST inhibitors, it is vital to unravel the key interactions between RmGST and GTX. To obtain molecular insights, molecular dynamics (MD) simulations were performed. MD simulations have been successfully used to reveal the behaviors of other tick proteins [33,34,35]. The key interactions for GSH and GTX binding are extracted here. Moreover, the structural and dynamic differences between GSH and GTX binding are also revealed. This insight is expected to provide a better understanding of an important insecticide-resistance mechanism, which may in turn facilitate the development of novel approaches to tick control.

## 2. Results and Discussion

### 2.1. Structural and Dynamic Properties of RmGST

**Figure 2:**
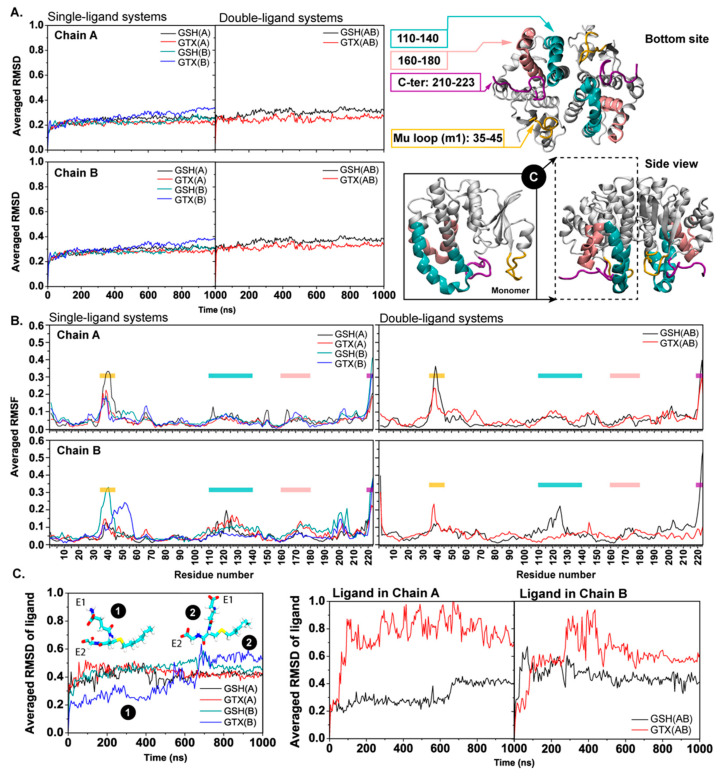
(**A**) C-alpha RMSDs of all systems (**left**) and a RmGST cartoon view with labelled highly flexible regions on the right (residues 35–45 (m1 loop), 110–140, 160–180, and C-terminal in yellow, cyan, pink, and purple, respectively). (**B**) RMSFs of each RmGST. (**C**) RMSDs of both GSH and GTX in single- (**left**) and double- (**right**) ligand systems. The large change (stages “1” to “2”) in GTX(B) orientation is shown as insets.

To explore how the ligand binding influences the RmGST flexibility, the C-alpha root mean square deviations (RMSDs) and root mean square fluctuation (RMSFs) were computed. The C-alpha RMSDs of whole RmGST were in the range of ~0.2–0.35 nm (Figure 2A). In single-substrate systems, almost all systems showed a comparable degree of RMSDs; however, GTX(B) displayed slightly higher structural fluctuation due to an increase in RMSD (Figure 2A). For full ligand occupancy (GSH(AB) and GTX(AB)), no significant differences in RMSDs between either substrates were captured, although GSH(AB) showed slightly structural flexibility (Figure 2A). Furthermore, the origin of structural flexibility was also investigated via RMSFs in Figure 2B. It appears that the protein dynamics was originated from the bottom part of RmGST, especially the mu loop (m1) (Figure 2A; right). This finding is in a good agreement with previous ligand-free RmGST study and crystallographic work [35,36]. However, it was observable that the helical region (residues 43–60) connecting the m1 loop with the core in chain B was also flexible in GTX(B) (Figure 2B). This high fluctuation is due to its displacement. More details are discussed later in the text. In addition, principal component analysis (PCA) and B factors were calculated to confirm the protein dynamics (Figure S1 in Supplementary Information). Only the motion obtained from the first principal component 1 (PC1) was considered here, because the first principal component 1 (eigenvector) accounts for major motions (Figure S1 in Supplementary Information). PCA clearly confirmed the high mobility of the bottom part of RmGST, especially the m1 loop (Figure S1 in Supplementary Information). Furthermore, the ligand flexibility was also determined in Figure 2C. In the case of single-ligand systems, both GSH and GTX induced a similar degree of ligand flexibility, except GTX(B) where the drift of RMSD was obtained (stages “1” to “2” in Figure 2C). Such shifted RMSD was because of the high mobility of the E1 end of GTX(B)’s peptide core (insets in Figure 2C). Seemingly, the increase in RMSD of GTX(B) as seen earlier (RMSD at ~500–600 ns) was due to the conformational change of the GTX substrate (RMSD at ~500–600 ns) (Figure 2A,C). However, the significant difference in structural mobility was captured in the double-ligand systems (GSH(AB) and GTX(AB)) (on the right in Figure 2C). GTXs in both chains showed higher flexibility than GSHs (Figure 2C). This indicates more mobility of bound GTX in double-ligand systems. This may be due to a presence of a hexyl chain on GTX. Further details will be discussed later in the text.

**Figure 3:**
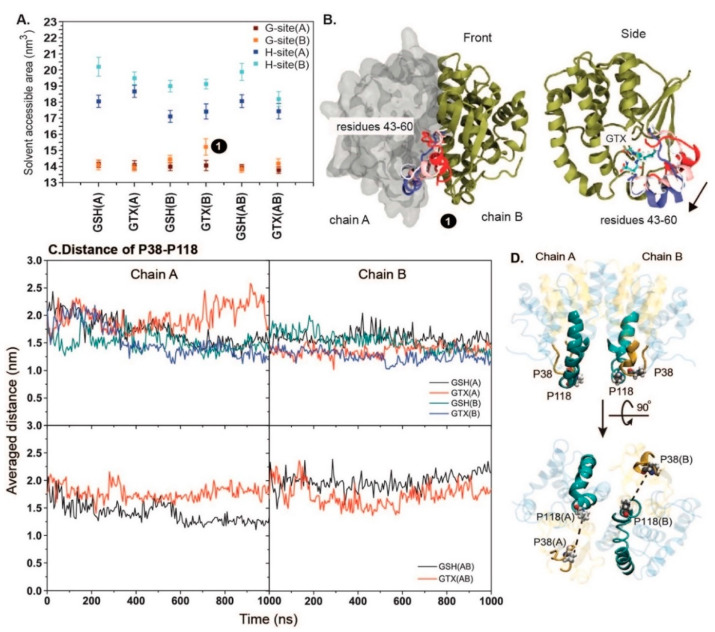
(**A**) Solvent accessible surface area (SASA) of G- and H- sites in all systems. G-site is defined by Y7, W8, W46, K50, N59, L60, Q72, and S73, while H-site includes A105, R108, M109, V112, R113, Y116, M163, Q166, L208, N209, and G210 [39]. The number “1” refers to the G-site cavity that is deviated from the others in GTX(B). The large movement of residues 43–60 causing the enlargement of G-site cavity in GTX(B) is shown in (**B**). The trajectory of the movement is shown in RWB format. The arrow indicates the direction of helix movement. (**C**) Distance between P38 and P118 on each subunit in all systems. A left column belongs to chain A, while chain B is shown on the right. (**D**) Displays the locations of P38 (on the m1 loop) and P118 on the protein core.

Furthermore, the effect of bound ligands on the pocket cavity size is determined via solvent accessible areas (Figure 3A). It can be seen in Figure 3A that the G-site (~14 nm^3^) is smaller than the H-site (~17–20 nm^3^). The binding of ligand results in the consistent size of the G-site pocket. Nonetheless, the significant expansion of the G-site was found in chain B of GTX(B) (number “1” in Figure 3A). This is due to the downward movement of the helical region (residues 43–60) (Figure 3B). Thus, this explains the high RMSF observed in Figure 2B. The movement of this helical region was consistent in both repeats of GTX(B). Such motion seems to be due to the reorientation of GTX as reported in Figure 2C. In contrast to the G-site, each subunit provides different volumes of H-site. The cavity size of the G-site is smaller and more preserved than that of H-site. Almost all systems have a comparable degree of G-site volume, but the sizes of the H-site between chains are different. Chain B contains a larger H-site than chain A in all cases (Figure 3A). As seen in many crystallographic studies [19,20,36], GTXs in each subunit are bound with different conformations. This may be due to the variability in the size of the H-site between subunits which allows various structural rearrangements. Furthermore, the difference in H-site volumes reflects the non-simultaneous acceptance of ligand between chains. Furthermore, this size deviation also suggests the tight binding of xenobiotic to RmGST may be based on the induced-fit mechanism as seen in plant phi-, and human pi-class GSTs [37,38]. Further experiment is required. In addition, the effect of the bound ligand on the dynamics of the m1 loop was also investigated by the distance between P38 at the tip of the m1 loop and P118 on the protein core (Figure 3C,D). Approximately, P38–P118 distances are ranged between 1.5 and 2.0 nm in all cases (Figure 3C,D). Comparing between two subunits, the more fluctuated P38–P118 distances observed in chain A indicate the more flexibility of the m1 loop in chain A (Figure 3C). Especially, the binding of one ligand (GSH(A), GTX(A), GSH(B), and GTX(B)) enhances more loop flexibility in chain A, especially GTX(A) and GTX(B) (a top left figure in Figure 3C). The result reflects the different pocket environments between chains which can lead to the dissimilar ligand-binding affinity and consequentially enzymatic activity between subunits. The difference in structural dynamics between subunits observed here are also observed in human and avian μ-class GSTs [19,36].

### 2.2. Influence of Bound Ligand on an RmGST Dimer

**Figure 4:**
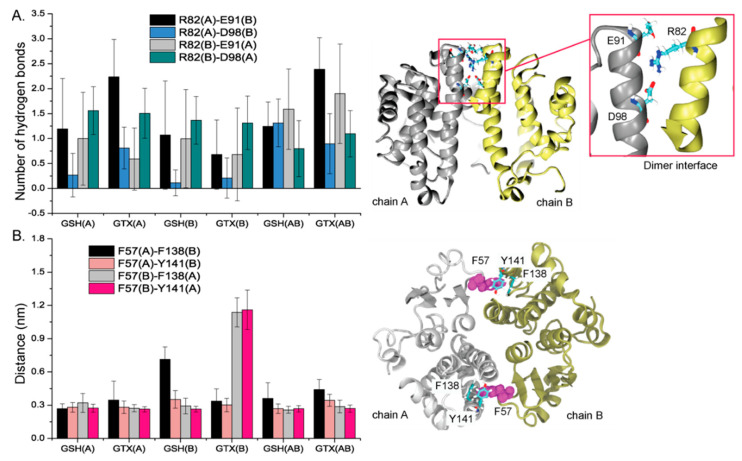
(**A**) Number of hydrogen bonds between residues located at a dimer interface where their locations are shown on the right. A red box indicates residues at a dimer interface. (**B**) Distances of F57 with F138 and F141 where their locations are displayed on the right. The silver and yellow structures display chains A and B with F57 in a magenta vdw surface.

The influence of bound substrate on dimerization is also investigated via polar inter-hydrogen bonds (R82–E91 and R82–D98 interactions) at the dimer interface (Figure 4A) and the lock-and-key structure. This lock-and-key motif is one of key characteristics for a-, μ-, and p-class GST dimers [26]. Herein, F57 (the “key”) in one subunit was wedged into a hydrophobic pocket of the other unit formed by F138 and Y141 (the “lock”) (Figure 4B). In Figure 4A, it appears that mono and di ligands induced the difference in inter-subunit interactions. All residues at the interface appeared to bind tightly in GSH(AB) and GTX(AB) (Figure 4A). Most R82(A)–D98(B) interactions seem to be weakened in mono-ligand systems (Figure 4A). For the lock-and-key feature, the existence of bound substrates in both mono- and di-ligand systems has no significant impact on the lock-and-key structure in most cases, except GSH(B) and GTX(B) (Figure 4B). F57(A)–F138(B) interaction in GSH(B) was slightly extended. The clear elongated distances of F57(B)–F138(A), and F57(B)–Y141(A) in Figure 4B are due to the downward movement of helical region (residues 43–60) (Figure 3B) which allows F57 to be reallocated and swipe away from the lock motif. 

### 2.3. RmGST-Ligand Interaction Networks

To investigate the behavior of all substrates, the average number of hydrogen bonds between each substrate and its environment were computed (Table 1). Seemingly, GSH seems to form more hydrogen bonds with RmGST than GTX. GSH in mono-ligand systems employs ~7–8 hydrogen bonds to be stabilized inside a pocket, whereas GTX requires only ~6–7 protein contacts (Table 1). Although GSH forms more interactions with RmGST, both GSH and GTX have a similar degree of water exposure (~9–11 water contacts). GTX also shows lower binding ability to RmGST in double-substrate systems (GTX(AB)) (Table 1). A number of GTX-RmGST interactions in GTX(AB) are dramatically reduced (~4–6 protein contacts) causing more water accessibility (~14–15 water contacts) (Table 1). This permits GTX to be more mobile inside a pocket. Unlike GTX, GSHs in GSH(AB) can maintain their interactions with RmGST which can enhance the structural rigidity of bound GSH. These results also demonstrate RmGST prefers GSH to GTX.

**Figure 5:**
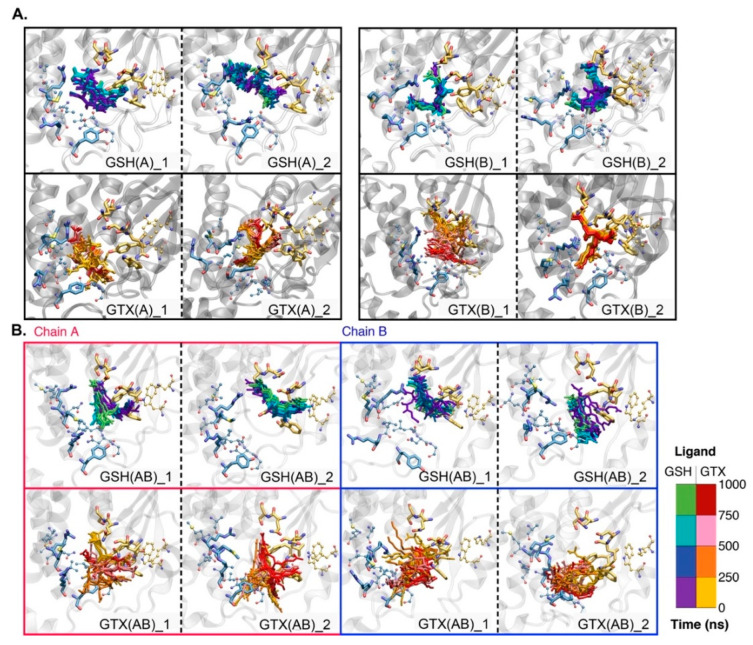
Ligand conformational change as a function of time in single-ligand (**A**) and double-ligand (**B**) systems. Red and blue boxes refer to chains A and B in GSH(AB) and GTX(AB). Residues in H- and G- sites are displayed in cyan and yellow licorice. All residues in CPK format refer to residues in the binding pocket.

**Figure 6:**
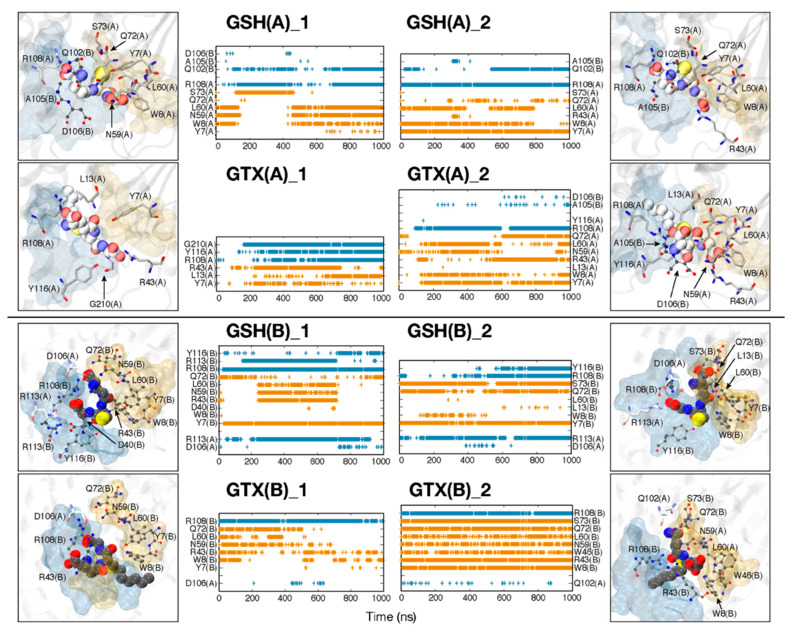
Occurrence of hydrogen bonds between RmGST and each substrate (GSH; top and GTX; bottom) in single-ligand systems (GSH(A), GSH(B), GTX(A), and GTX(B)). Yellow and cyan bands refer to hydrogen bonds formed by residues in G- and H- sites, respectively. The locations of ligand-binding residues are shown in boxes (top view). Residues in a licorice format belong to chain A, while those in chain B are labelled in CPK.

**Figure 7:**
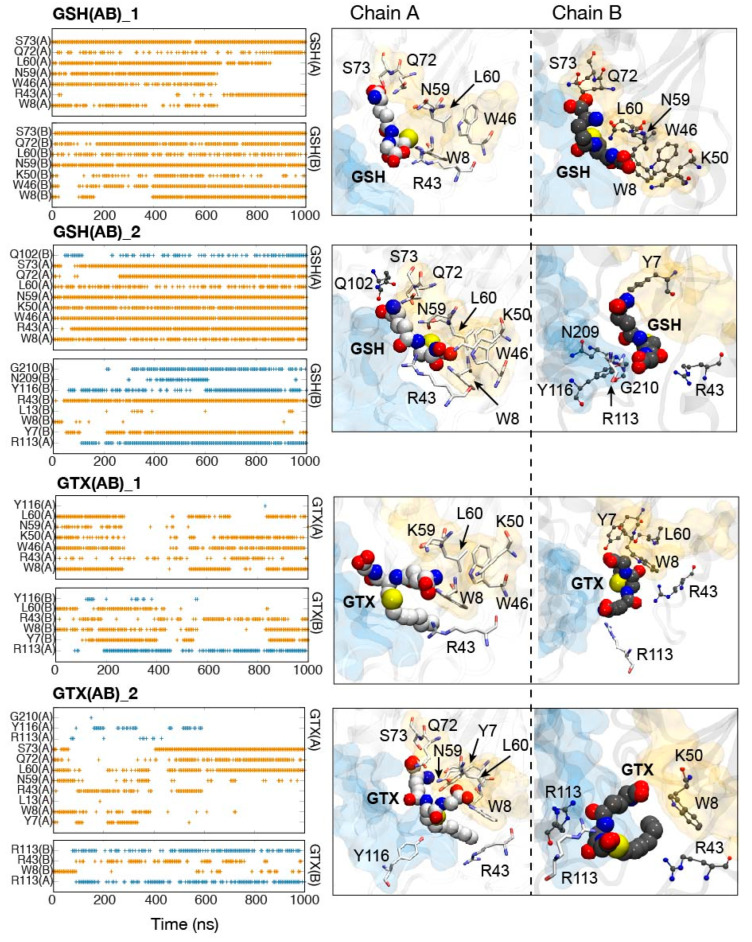
Occurrence of hydrogen bonds between RmGST and each substrate. Yellow and cyan bands refer to hydrogen bonds formed by residues in G- and H- sites, respectively. The locations of ligand-binding residues are shown in boxes. Residues in a licorice format belong to chain A, while those in chain B are labelled in CPK.

In Figure 5, the orientations of each substrate as a function of time are presented. The dynamics of GSH and GTX in Figure 5 are in a good agreement with the hydrogen bond analysis (Figure 6 and Figure 7). More GSH-RmGST interactions allow GSH to be more rigid inside a pocket, whereas GTX with lower protein contacts induce more ligand mobility (Figure 5A,B). Except GTX(B)_2, various conformations of bound GTX were observed (Figure 5). GTX seems to interact with both residues in G- and H- sites, while GSH prefers to form contacts with G-site-lining residues (Figure 5). It was noticeable that GTXs in GTX(AB) were more mobile due to less protein contacts (Table 1 and Figure 5B). Such high mobility of GTX supports the higher RMSDs reported earlier.

Furthermore, the key interaction networks for ligand binding were investigated (Figure 6 and Figure 7). Considering one-substrate systems (Figure 6), the binding of both GSH and GTX requires cooperation between subunits (Figure 7). Both ligands can interact with residues from both chains, thus this highlights the importance of being a dimer for RmGST function. As reported earlier that each chain provides different environments in the active site, the interaction networks in each subunit were thus non-identical (Figure 6 and Figure 7). However, the GSH poses between chains were quite consistent, whereas GTX in each chain oriented in a disparate direction (Figure 5, Figure 6 and Figure 7 and Figure S2 in Supplementary Information). The reorientation of E1 and E2 tails were the root of multiple GTX conformations observed here (Figure 1B and Figure S2 in Supplementary Information). The variability of GTX orientations was also captured in RmGST-GTX crystal structures from other μ-class GSTs [19,36]. Although the shift of polar E1 and E2 termini induces the GTX conformational change, all s-hexyl moieties point toward the connecting loop (residues 8–11) between strand β1 and helix α1 in all cases (Figure 1A and Figure S3 in Supplementary Information). This hexyl chain protrudes into a pocket and stays stably by W8, I10, C35, Y116, and G210 (Figure S4A in Supplementary Information). This protrusion was also found in other GTX-GST crystal structures [20,21,36]. Our findings also suggest the permanent protrusion of this hexyl group to the H-site. This penetration can interfere with the binding of co-substrates such as acaracides. Moreover, the high mobility of E1 and E2 tails on GTX allows a range of RmGST-GTX interactions. In the case of single-GTX systems, GTX can mainly hydrogen bond with Y7, W8, R43, N59, L60, and R108, where a wide range of GTX conformations were found (Figure 6). Unlike other GTX systems, GTX(B)_2 showed high rigidity due to the permanent interactions with W8, R43, W46, N59, L60, Q72, S73, and R108 (Figure 6 and Figure S4B in Supplementary Information). For double-GTX systems, although each subunit provides different interaction networks for GTX binding, a similar set of main interactions to single-GTX systems is formed (interactions with Y7, W8, R43, N59, L60) (Figure 7). No GTX-R108 interaction was found in GTX(AB), but the additional hydrogen bond with R113 was identified (Figure 7). Each chain also induced different GTX conformations. This suggests each chain functions alternatingly. Moreover, the high GTX flexibility and the protrusion of its hexyl moiety to the H-site reported earlier also suggest the dual role of GTX in preventing the conjugation reaction and the binding of acaracide. Further experimental study is required to prove this hypothesis. 

In the case of GSH, it is interesting that the presence of two GSHs induced the tighter binding to RmGST. Although a small dissimilar number of RmGST-GSH interactions were spotted, GSH seemed to be more rigid inside a pocket when compared with GTX. For GSH, the E2 tail directs to the tip of the mu loop and mainly interacts with residues in the G-site (W8, R43, W46, K50, L60). When GSH moves towards the H-site, this allows the E2 terminus to hydrogen bond with Q102, R113, Y116, N209, G210 (Figure 6 and Figure 7). In the case of the E1 end, it was lifted toward the dimer interface and became stabilized mainly by N59, Q72, and S73 (Figure 6 and Figure 7, and Figure S2 in Supplementary Information). The GSH conformation is likely to be preserved, but it was still able to float inside a pocket due to the large, connected H- and G- sites. However, most GSH simulations demonstrate GSH prefers to stay in the G-site. In human and rat, Y6 contributes to the stabilization of the thiol group [40], whereas Y115 involves the addition of GSH to xenobiotic and product release [41]. These residues are also conserved in RmGST (Y7 and Y116). Only the contribution of Y7 to substrate binding is studied. For GSH, the hydroxyl group of Y7 was in close proximity (distance ≤ 0.5 nm) to a sulfur atom (S) on GSH in all cases, whilst the S atom on GTX shifted away from OH on Y7 in double-ligand systems (distance of ~0.6–1 nm in Figure S5 in Supplementary Information). The shift in Y7-S distance in GTX(AB) implies the altered environment in the active site which could interfere with further xenobiotic binding. Unlike GTX, the higher rigidity of the GSH core permits Y7 to interact with the -SH in all cases. This frozen GSH arrangement can accommodate the easy binding of pesticides and sequential conjugation reaction.

## 3. Materials and Methods

### 3.1. RmGST-Ligand Complex Preparation

The three-dimensional structure of RmGST modelled by MODELLER [42] from a previous work was used as a starting structure [35]. The good structural quality had be confirmed and compared to the template using SAVES server [43] (Figure S6 in Supplementary Information ). Glutathione (GSH) and s-hexyl glutathione (GTX) structures were obtained from previous crystallographic studies (PDB codes: 1XW6 (GSH) [36] and 1GSU (GTX) [44]). The ligand parameters were constructed using Antechamber via Acpype server [45,46,47,48,49]. For each system, the initial ligand-RmGST structure was obtained by superimposing RmGST to the GST-ligand crystal structures (PDB codes: 1XW6 (GSH) and 1GSU (GTX)). A ligand protein was placed in a cubic box (with a dimension of 5 × 5 × 5 nm^3^) and solvated with TIP3P water molecules (~22,500 molecules). The protonation states of charged amino acids were set at physiologic pH. Counter ions were added to neutralize the system. The energy minimization of 50,000 steps was performed to remove close contacts, using steepest descent algorithm followed by 1-ns NVT and 10-ns NPT runs. To explore the effect of single and double ligand binding, the systems with one ligand in one chain (GSH(A), GTX(A), GSH(B), and GTX(B)) and with one ligand in each chain (GSH(AB) and GTX(AB)) were set. The prefixes of “GSH” and “GTX” were used to represent the systems with GSH and GTX, respectively. The letter in a bracket ((A) and (B)) indicates the ligand-containing chain. Two copies with different random seeds of 1000 ns production runs were performed. The endings of “1” and “2” refer to simulations 1 and 2. In sum, 12 systems were set here (Table 2). The data shown are the average between the two simulations.

### 3.2. Simulation Protocol

The GROMACS 5 package (www.gromacs.org (accessed on 16 June 2021)) [50] was employed with Amber99SB-ILDN force fields. The particle mesh Ewald (PME) techniques [51] with a Fourier spacing of 0.12 nm and a short range cut-off of 1 nm were used for electrostatic treatment. The simulations were conducted in the constant number of particles, pressure, and temperature (NPT) ensemble. The Berendsen algorithm at 1 bar with a coupling constant τ_p_ = 1 ps was used for pressure coupling. The temperature of the protein, ligand, and solution were coupled separately using the v-rescale thermostat [52] at 300 K with a coupling constant τ_t_ = 0.1 ps. The time step of 2 fs was used for integration. The coordinates were recorded every 2 ps. 

All data were analyzed by GROMACS tools and in-house codes. “Gmx hbond” was used to compute all hydrogen bonds where the hydrogen-donor-acceptor cutoff angle was set to 30° and the cutoff radius (X-acceptor) was 0.35 nm. Root mean square deviation (RMSD) and root mean square fluctuation (RMSF) were calculated using an initial structure at 0 ns from each production run as a reference. A principal component analysis (PCA) was calculated using “gmx covar” and “gmx anaeig”. VMD was used for visualization and graphic images [53].

## 4. Conclusions

In this study, the interaction modes between RmGST and its substrate (GSH) and inhibitor (GTX) were revealed through molecular dynamics simulations. GSH was more rigid inside the active site, while GTX was flexible. GTX occupied both active sites (H- and G- sites), while GSH mostly aligned in the G-site and left the free H-site for further binding of co-substrate. The also suggests the binding order where GTX/GSH acts as the first substrate and xenobiotic or insecticide comes second. Further experiment is needed. Comparing the binding between GSH and GTX, GSH seemed to be more rigid inside the G-site which may facilitate further conjugation reaction. The presence of an s-hexyl chain on GTX seemed to significantly disrupt the interaction network inside a pocket. Although the hexyl moiety occupied the H-site close to the connecting loop between α1 and β1 similar to other existing GST-GTX crystal structures [19,36], this additional long hexyl chain shifted the E1 and E2 chains away from the binding site causing the loss of main interactions with R43, Q72, and S73. This led to the increased flexibility of E1 and E2 tails resulting in the multiple GTX orientations inside a pocket. Moreover, the existence of the aliphatic hexyl inside the H-site may block the xenobiotic binding. This finding can be used to explain why GTX remains the effective inhibitor used for metabolic resistance in insects [20]. The insights obtained here could provide the basis for the discovery and optimization of new potential RmGST inhibitors.

## Figures and Tables

**Table 1:** Average number of hydrogen bonds with standard deviation of each ligand with RmGST and water.

System	Run	Number of Hydrogen Bonds			
		Protein-Ligand		Ligand-Water	
		Chain A	Chain B	Chain A	Chain B
GSH(A)		8.35 ± 0.65		11.51 ± 1.81	
GSH(B)			7.52 ± 0.88		9.94 ± 2.09
GSH(AB)	1 2	7.14 ± 1.44 10.35 ± 1.65	8.47 ± 1.33 6.59 ± 1.51	12.23 ± 1.87 9.01 ± 1.84	10.48 ± 2.10 12.87 ± 2.26
GTX(A)		7.06 ± 0.96		11.05 ± 2.31	
GTX(B)			6.48 ± 0.97		10.04 ± 1.97
GTX(AB)	1 2	4.53 ± 1.39 3.92 ± 1.21	4.94 ± 1.59 6.37 ± 0.87	14.59 ± 2.44 15.09 ± 2.38	14.14 ± 2.56 13.50 ± 2.29

**Table 2:** All systems set in this study.

Ligand	System	Time (ns)	No. of Simulations (1000 ns)
Glutathione (GSH)	GSH(A)	1000	2 (GSH(A)_1 and GSH(A)_2)
	GSH(B)		2 (GSH(A)_1 and GSH(A)_2)
	GSH(AB)		2 (GSH(AB)_1 and GSH(AB)_2)
s-Hexyl glutathione (GTX)	GTX(A)	1000	2 (GTX(A)_1 and GTX(A)_2)
	GTX(B)		2 (GTX(B)_1 and GTX(B)_2)
	GTX(AB)		2 (GTX(AB)_1 and GTX(AB)_2)

## Data Availability

The data of current study are available from the corresponding author on reasonable request.

## Supplementary Materials

The supporting information can be downloaded at: https://www.mdpi.com/article/10.3390/ijms232112775/s1.

## Abbreviations

GST	Glutathione S-transferase
GSH	Glutathione
GTX	S-hexylglutathione
MD	Molecular Dynamics
