# Supplementary material for: Observing How Glutathione and S-Hexyl Glutathione Bind to Glutathione S-Transferase from Rhipicephalus (Boophilus) microplus
Source: Int J Mol Sci. 2022 Oct 23;23(21):12775. doi: 10.3390/ijms232112775 (PMC9655991; doi:10.3390/ijms232112775)
Supplement: Supplementary file 1 [file ijms-23-12775-s001.zip › ijms-1973623-supplementary.pdf]

Supplementary Information

Observing how glutathione and s-hexyl glutathione bind to Glutathione S-transferase from *Rhipicephalus (Boophilus) microplus*

Warin Rangubpit [1,2,3], Eukote Suwan [1,2], Danai Sangthong [1,2], Kannika Wongpanit [4], Roger W. Stich [5], Prapasiri Pongprayoon [3,6,*] and Sathaporn Jittapalapong [1,2,*]

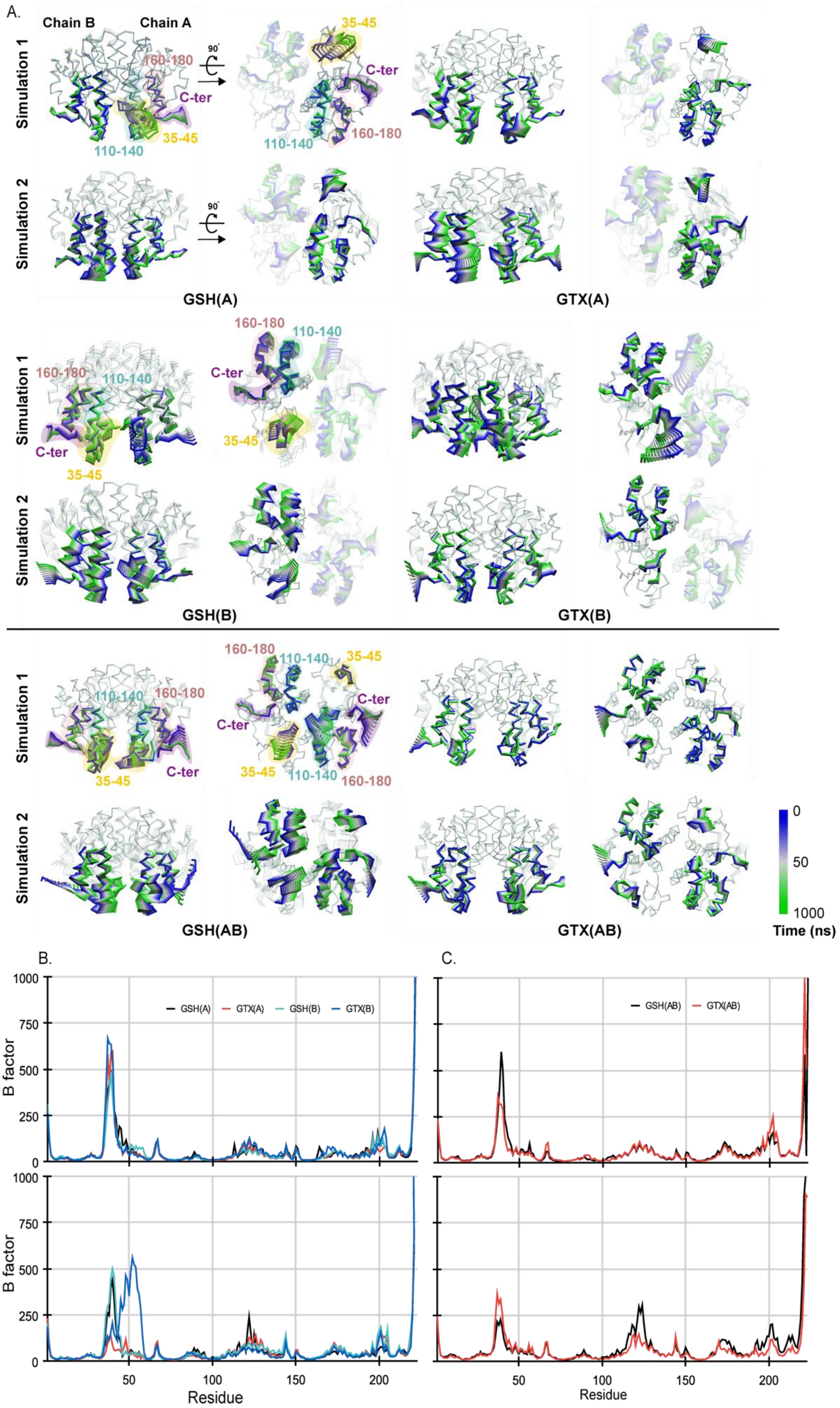

Figure S1 (A) Principal Component Analysis (PCA) of C-alpha structure of GST. (B) and (C) B factors of each system.

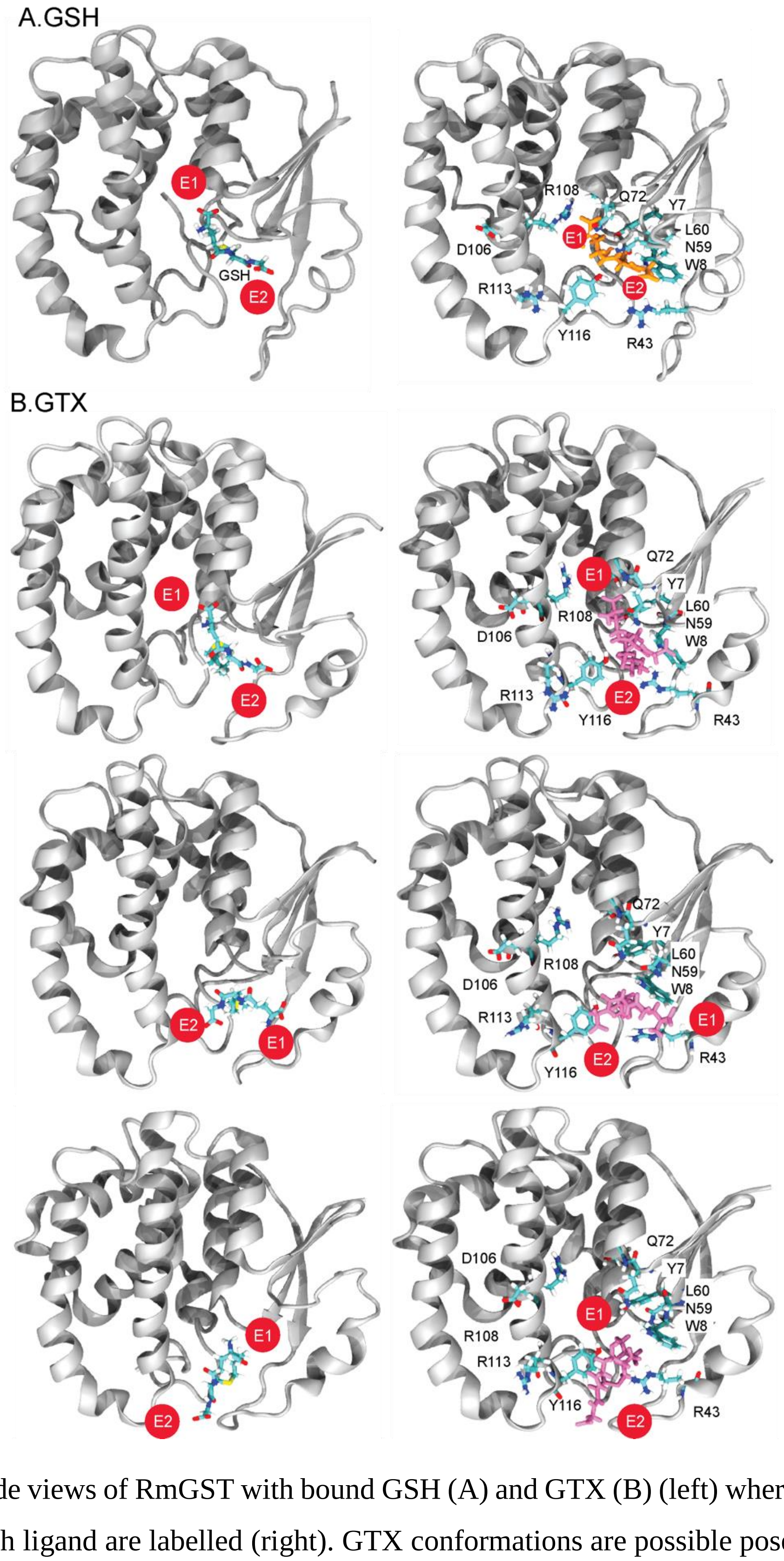

Figure S2. Side views of RmGST with bound GSH (A) and GTX (B) (left) where key residues binding to each ligand are labelled (right). GTX conformations are possible poses found in all GTX simulations. Orange and pink structures are GSH and GTX.

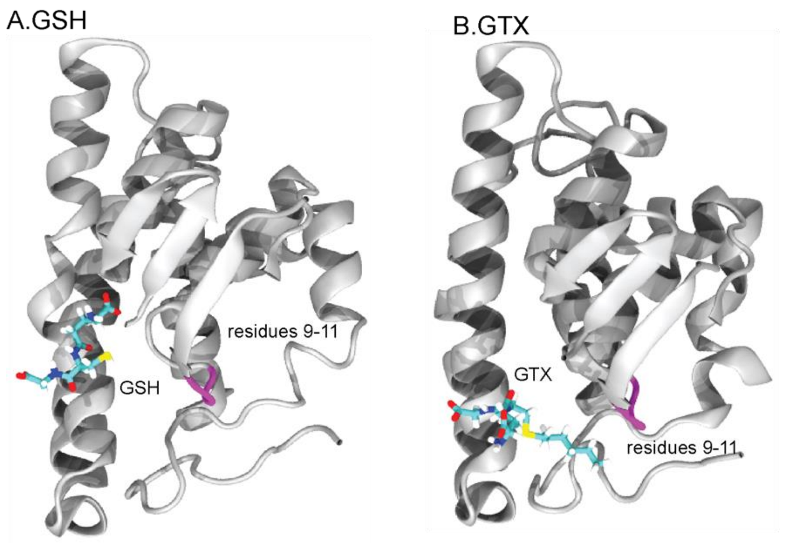

Figure S3. Front views of RmGST with bound GSH (A) and GTX (B). The connecting loop between B1 and A2 is labelled in magenta.

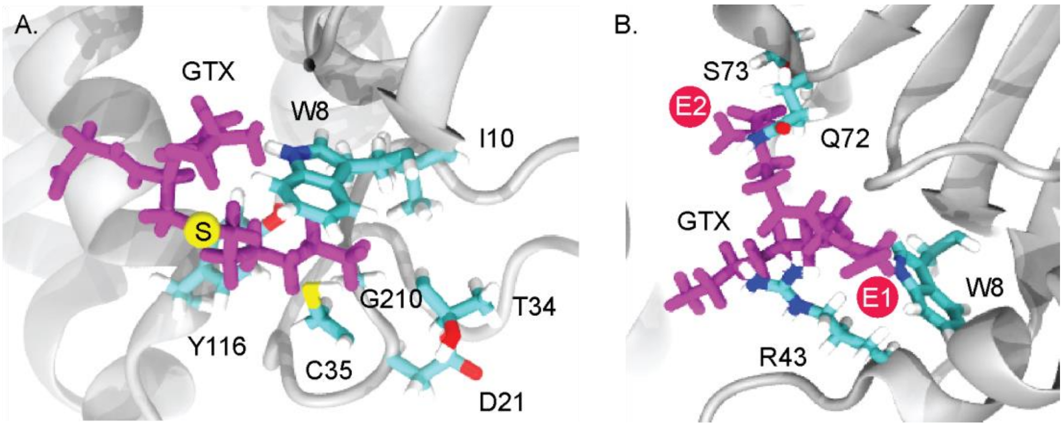

Figure S4. (A) Orientation of S-hexyl moiety of GTX in a pocket. A Sulphur atom on GTX is shown as a yellow bead. (B) Orientation of GTX in GTX(B)_2.

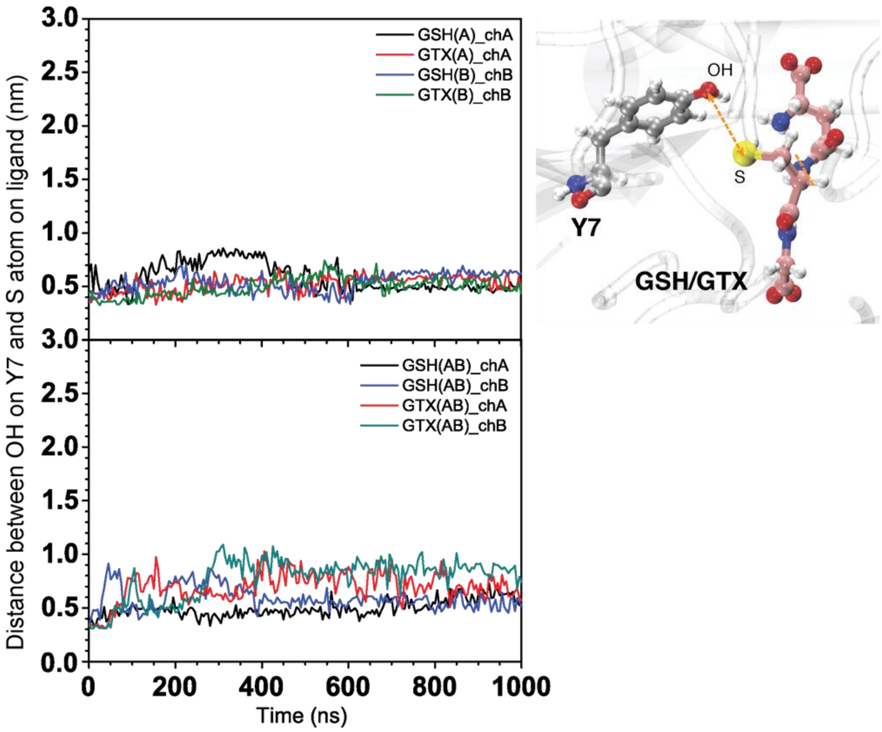

Figure S5. (A) Distances between OH group on Y7 and Sulphur atom on GSH/GTX substrates in all systems. The location of each component is shown on the right.

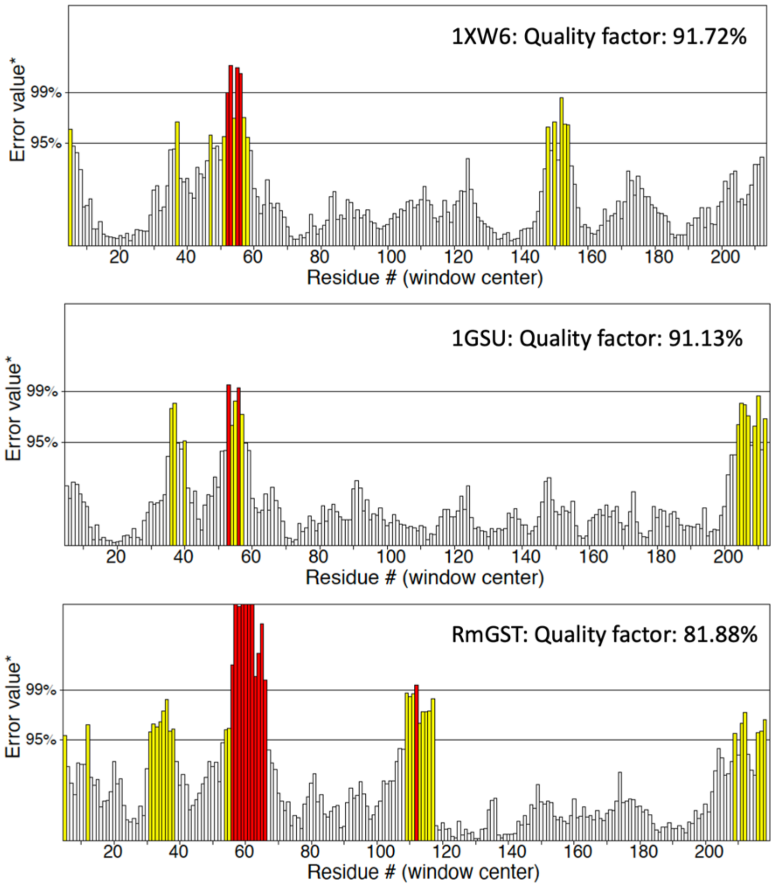

Figure S6 ERRAT score of human GST (PDB code: 1XW6), avain GST (PDB code: 1GSU), and RmGST structures.
